# Rotten Hazelnuts Prediction *via* Simulation Modeling—A Case Study on the Turkish Hazelnut Sector

**DOI:** 10.3389/fpls.2022.766493

**Published:** 2022-04-04

**Authors:** Taynara Valeriano, Kim Fischer, Fabrizio Ginaldi, Laura Giustarini, Giuseppe Castello, Simone Bregaglio

**Affiliations:** ^1^Council for Agricultural Research and Economics (CREA), Research Centre for Agriculture and Environment, Bologna, Italy; ^2^Ferrero Hazelnut Company, Ferrero Trading Lux S.A., Senningerberg, Luxembourg; ^3^RSS-Hydro SARLS, Dudelange, Luxembourg; ^4^SOREMARTEC ITALIA S.r.l., Alba, Italy

**Keywords:** simulation model, decision support system, rotten hazelnut, sensitivity analysis, automatic calibration

## Abstract

The quality defects of hazelnut fruits comprise changes in morphology and taste, and their intensity mainly depends on seasonal environmental conditions. The strongest off-flavor of hazelnuts is known as rotten defect, whose candidate causal agents are a complex of fungal pathogens, with *Diaporthe* as the dominant genus. Timely indications on the expected incidence of rotten defect would be essential for buyers to identify areas where hazelnut quality will be superior, other than being useful for farmers to have the timely indications of the risk of pathogens infection. Here, we propose a rotten defect forecasting model, and we apply it in the seven main hazelnut producing municipalities in Turkey. We modulate plant susceptibility to fungal infection according to simulated hazelnut phenology, and we reproduce the key components of the *Diaporthe* spp. epidemiological cycle *via* a process-based simulation model. A model sensitivity analysis has been performed under contrasting weather conditions to select most relevant parameters for calibration, which relied on weekly phenological observations and the post-harvest analyses of rotten incidence in the period 2016–2019, conducted in 22 orchards. The rotten simulation model reproduced rotten incidence data in calibration and validation datasets with a mean absolute error below 1.8%. The dataset used for model validation (321 additional sampling locations) has been characterized by large variability of rotten incidence, in turn contributing to decrease the correlation between reference and simulated data (*R*^2^ = 0.4 and 0.21 in West and East Black Sea region, respectively). This denotes the key effect of other environmental and agronomic factors on rotten incidence, which are not yet taken into account by the predictive workflow and will be considered in further improvements. When applied in spatially distributed simulations, the model differentiated the rotten incidence across municipalities, and reproduced the interannual variability of rotten incidence. Our results confirmed that the rotten defect is strictly dependent on precipitation amount and timing, and that plant susceptibility is crucial to trigger fungal infections. Future steps will envisage the application of the rotten simulation model to other hazelnut producing regions, before being operationally used for in-season forecasting activities.

## Introduction

Crop quality is the major determinant of the economic and nutritional value of agricultural products, as it influences their purchase attractiveness by consumers and their acceptability to buyers (Cappelli et al., 2014; Melovic et al., 2020). Quality defects induce indirect yield losses, as the consequence of non-compliance with required quality standards (Battilani et al., 2018), which are needed to gain a competitive advantage in the domestic and export market. In the case of hazelnuts, high quality in-shelled fruits are increasingly requested by the confectionary industry (Cristofori et al., 2008), especially from Turkey, the world leader of production and export (FAO, 2019). However, hazelnut fruits are often affected by quality defects associated with off-flavors (Pscheidt and Ocamb, 2017), which decrease their usability in industrial products. The detection of externally visible and asymptomatic defects after kernel cutting is one of the main determinants of hazelnut quality (Battilani et al., 2018).

We focus here on the rotten defect, the strongest sensory off-note of hazelnut fruits, which annually threatens kernel availability and marketability (Arciuolo et al., 2020). The term “rotten hazelnuts” derives from the industrial jargon and refers to fruits with necrotic spots and/or internal browning, resulting in a black kernel in the worst case. The incidence of rotten defect on harvested nuts usually fluctuates in the range of 1–15% (Arciuolo et al., 2020), but even a small presence of damaged fruits could be detrimental for organoleptic properties. So far, few studies focused on the identification of the causes of hazelnut rotten defect and the etiological agents have not been unanimously defined yet. Battilani et al. (2018) conducted a 4-year experimental study in the Caucasian region, concluding that *Diaporthe* was the dominant genus in defected kernels, among many other fungal species associated with rotten hazelnuts (i.e., *Alternaria* spp., *Cladosporium* spp., *Fusarium* spp., and *Colletotrichum* spp.). Further, same authors observed a positive correlation between the precipitation amount during the growing season and the incidence of rotten defect.

Process-based simulation models are needed to extrapolate the experimental results from one site to another, thus enabling the development of early warning systems, to either optimize the chemical control of plant diseases or perform scenario analyses on pathogen suitability over large areas (Gillespie and Sentelhas, 2008). In addition, plant disease models are increasingly requested by private and public stakeholders to timely identify critical situations and quantify the expected impacts on yield and quality (Bregaglio et al., 2016; Valeriano et al., 2021). With these premises, we developed a new simulation model to predict the incidence of the rotten defect on hazelnuts. We followed the underlying hypothesis that *Diaporthe* spp. are the main causal agents of rotten hazelnuts; however, the new model is composed by generic sub-models, which can be distinctly parameterized according to thermal and moisture requirements of different fungal pathogens. A sensitivity analysis was performed to gain insights into the model plasticity across contrasting climatic conditions (Confalonieri et al., 2012) and to highlight the most relevant parameters to be adjusted to increase the prediction accuracy (Ruget et al., 2002; Vazques-Cruz et al., 2014). The model was then coupled with an automatic optimization tool, and key parameters were calibrated within their biological meaningful ranges to modulate the response of different epidemiological processes to environmental conditions (Angulo et al., 2013). The model evaluation has been performed with independent and additional field datasets from the seven main hazelnut producing municipalities in Turkey. This work lays the basis to set up a digital decision support system, enabling an early prediction of the environmental suitability of fungal pathogens associated with the occurrence of rotten hazelnuts. The fields of application of such a system comprise in-season prediction to timely identify areas where hazelnut quality is predicted to be higher.

## Materials and Methods

### Overview of the Study

**FIGURE 1:**
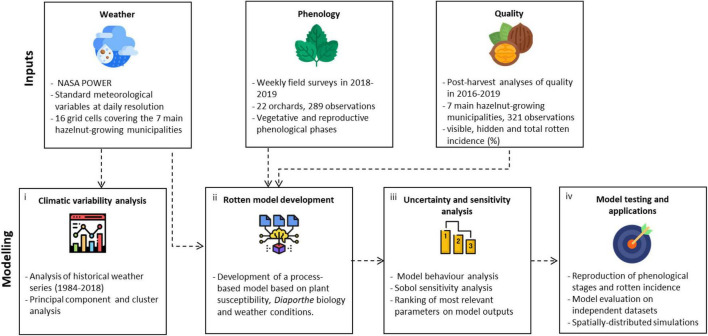
Workflow of the study. Input data sources (weather data, phenological observations, and rotten incidence data) were used (i) to perform a climatic characterization of the study area, (ii) to develop a process-based simulation model of rotten hazelnuts, which was then subjected to (iii) an uncertainty and sensitivity analysis to understand the model behavior across climatic conditions. Finally, (iv) the model was calibrated and evaluated using field data and applied over the Turkish hazelnut area.

The workflow of this study is articulated in four steps (Figure 1). Historical weather series (1984–2018) in the study area were processed to compute agrometeorological indices, which were used to identify the clusters of hazelnut growing seasons sharing similar climatic conditions *via* multivariate analyses (step 1). A process-based simulation model to estimate the incidence of rotten hazelnuts was developed using available knowledge on *Diaporthe* spp. (Emmett et al., 1992; Erincik et al., 2003; Anco et al., 2013): the main components of the epidemiological cycle were formalized in sub-models driven by hourly weather variables and biologically meaningful parameters (step 2). The new model was subjected to a global sensitivity analysis to identify the most relevant parameters in modulating the key outputs, also considering their uncertainty under contrasting climatic conditions from step 1 (step 3). The most relevant parameters were then calibrated using the ground truth data of rotten incidence from post-harvest analysis and phenological observations collected in 22 orchards; the model evaluation was carried out on independent and additional 321 datasets, before running spatially distributed simulations over the whole hazelnut producing area in Turkey (step 4).

### Input Data Sources for the Modeling Activities

**FIGURE 2:**
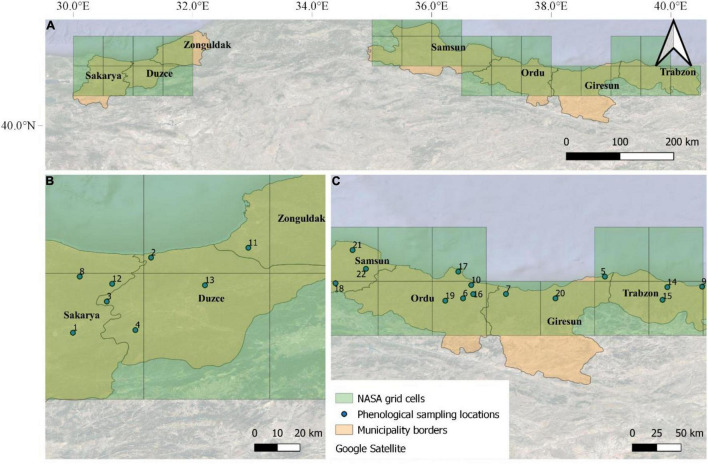
The seven main municipalities where hazelnuts are cultivated in Turkey **(A)**. The locations of the 22 orchards where phenological observations were performed on a weekly basis in the Western **(B)** and Eastern **(C)** Black Sea are overlapped with NASA-POWER grids (27 grid cells).

The input weather data for the modeling activities obtained from the National Aeronautics and Space Administration (NASA) Langley Research Center (LaRC) Prediction of Worldwide Energy Resource (POWER) Project funded through the NASA Earth Science/Applied Science Program, which provides daily meteorological variables at 0.5° × 0.5° resolution grid. We used here maximum and minimum air temperature (°C), dew point temperature (°C), relative humidity (%), and average wind speed (m s^–1^). Hourly air temperature was estimated from daily maximum and minimum air temperature, according to Campbell (1985), whereas air relative humidity was derived according to Bregaglio et al. (2010), based on the models proposed by Linacre (1992), Allen and FAO (1998), and Hahn et al. (1998). Hourly leaf wetness was estimated from hourly air temperature, dew point temperature, wind and relative humidity, according to Kim et al. (2002). In total, 27 NASA-POWER grid cells have been selected to cover the main hazelnut producing regions of Turkey (Figure 2).

The hazelnut rotten simulation model was calibrated using phenological observations and post-harvest rotten incidence data collected in 22 hazelnut orchards located in the municipalities of Samsun, Ordu, Giresun, and Trabzon (Eastern Black Sea area), and Duzce, Sakarya, and Zonguldak (Western Black Sea area) (Figure 2). Phenological observations were collected weekly in these orchards in the 2018 and 2019 growing seasons, whereas the post-harvest analyses of rotten incidence were carried out in the period of 2016–2019 (4 years). The model evaluation was carried out on 321 additional locations where post-harvest analyses on hazelnuts samples were available in the same reference period (2016–2019, as shown in Arciuolo et al., 2020, this special issue). In all datasets, hazelnuts were collected at full ripening (phase R13, as shown in Supplementary Table 2) on 100 trees per orchard (30 hazelnuts per tree), when average kernel humidity was about 10%, to obtain approximately 15 kg of hazelnuts sample^–1^ (about 3,000 hazelnuts). Hazelnut fruits were dried on pallets to facilitate the husk removal and mechanical dehusking; after a second drying period to ease the cracking procedure, they were manually shelled and observed for defects after cutting kernels in two halves. The percentage incidence of rotten hazelnuts with visible and invisible defects was determined in laboratory and used as reference data to evaluate model performances.

The 22 orchards where calibration activities were performed are located in 13 out of the 27 NASA grid cells covering the whole hazelnut producing area in Turkey. Therefore weather data from these 13 grid cells were used for model calibration. The model was then evaluated comparing simulation results obtained on all 27 grid cells with median rotten hazelnuts incidence from the additional 321 sampling locations, after averaging them based on the NASA-POWER grid cell they fall in.

### Model Development

Differently from other plant diseases whose etiological agent is well known, several genera (*Diaporthe*, *Alternaria*, *Cladosporium*, *Fusarium*, and *Colletotrichum*) are associated with rotten hazelnut defects. *Diaporthe* spp. emerged as candidate pathogens in the Caucasian region (Battilani et al., 2018). Based on this work, the rotten simulation model presented here is composed by two modules: a process-based model to simulate the epidemiological processes of the *Diaporthe* spp. cycle, coupled with the reproduction of the susceptibility of hazelnuts to fungal infections as modulated by their phenological development. The large uncertainty associated with the causal agent and the heterogeneity of *Diaporthe* strains isolated on rotten hazelnuts in Turkey (Arciuolo et al., 2020) led us to develop a hybrid approach, where the indicators of the suitability of weather conditions to generic fungal pathogens are derived from long-term simulations (climatic norm, 30 years over whole Turkey, 1988–2018).

**FIGURE 3:**
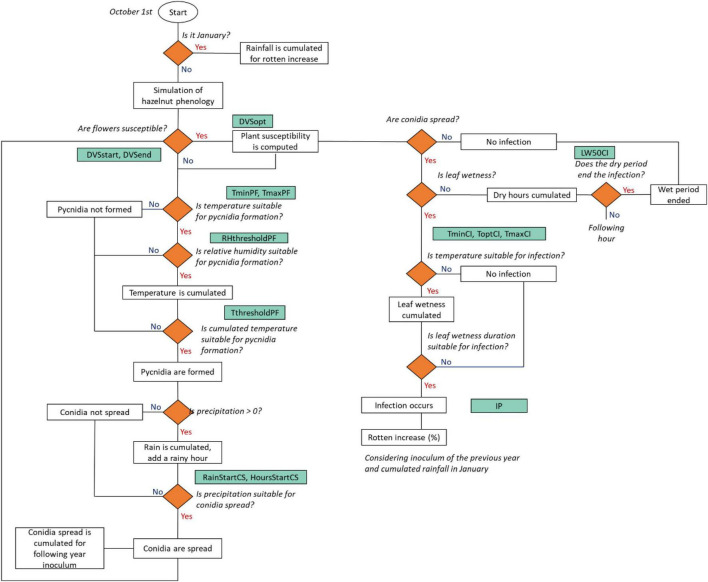
A flowchart of the rotten incidence simulation model. Decisions are indicated as rhombi, parameters are reported in green boxes and model outputs/events are indicated as white boxes. The acronym, description, and units of the parameters are reported in Table 2.

The workflow of the rotten hazelnut simulation model is presented in Figure 3. Model simulations start on October 1, defined as the starting date of the hazelnut growing season. The simulation of the hazelnut reproductive phases is performed according to Bregaglio et al. (2016, 2020, 2021), this special issue. Eight phenological phases were simulated for female reproductive development, from flowering to nut dropping. The simulation of the suitability of weather conditions to rotten increase starts with the formation of pycnidia, i.e., suitable structures for the overwintering of the pathogen (Arciuolo et al., 2021), as driven by air temperature and relative humidity. When pycnidia are formed and mature, they produce cirrhi where conidia develop, i.e., the asexual form of the pathogen (Pscheidt and Pearson, 1991). The model considers that conidia can spread based on the rainfall intensity and duration. Once the plant susceptibility period is reached, the flowers are receptive for the fungal spores. Fungal infection is simulated when conidia are spread, with suitable leaf wetness duration and temperature. The simulated rotten incidence (%) is defined by the following variables: the number of hours suitable for infection events in the current season, the cumulated rainfall in January, and the number of hours suitable for conidia spread in the previous season, to consider the carry over effect of the inoculum load from 1 year to the next.

At each time step, when hourly air relative humidity is higher than a threshold (RHthresholdPC) and temperature is within the minimum and maximum for pycnidia formation (TmaxPF, TminPF), hourly temperature is cumulated. Once the cumulated hourly temperature (CumT, °C) reaches a given threshold (ThresholdPF), pycnidia start to form (*PycnidiaFormation*, 0-1, Eq. 1).


(1) PycnidiaFormation={1ifCumT≥ThresholdPF 0otherwise

Conidia are spread (*ConidiaSpread*, 0-1, Eq. 2) when both cumulated rainfall (CumHourlyRain, mm) and consecutive rainy hours (RainyHours, h) exceed a threshold (RainStartCS; HoursStartCS).


(2) $$ConidiaSpread=\left\{ \begin{array}{c} 1ifCumHourlyRain\geq RainStartCS\mathrm{and}\\ RainyHours\geq HoursStartCS\\ 0otherwise \end{array}\right.$$

The number of suitable hours for conidia spread (CumCS) is used as a proxy of the inoculum load for the following year and to modulate the increase of rotten incidence (%, *IncreaseR*, Eq. 3) associated with each infection event. *IncreaseR* depends on the maximum rotten incidence increase (IP, %), modulated by the normalization of CumCS according to the median of simulated hours when conidia were spread in the period 1984–2018 ($\bar{CS}$, Eq. 3).


(3) $$IncreaseR=IP*\left(1+\frac{CumCS-\bar{CS}}{\bar{CS}}\right)$$

The plant susceptibility (*PS*, 0-1, Eq. 4) is activated when female flowers are receptive for fungal spores (DVSstart). The codes and description of the phenological phases are reported in Supplementary Table 2. The optimum susceptibility and the end of the susceptible period are driven by the parameters DVSopt and DVSend.


(4) $$PS=\left\{ \begin{array}{c} 0\;if\;DVS_{start}<DVS>DVS_{end}\\ \left(\frac{DVS_{end}-DVS}{DVS_{end}-DVS_{opt}}\right)*{\left(\frac{DVS-DVS_{start}}{DVS_{opt}-DVS_{start}}\right)}^{\left(\frac{DVS_{opt}-DVS_{start}}{DVS_{end}-DVS_{opt}}\right)}otherwise \end{array}\right.$$

The infection events are simulated as a function of leaf wetness, which is cumulated (CumLW) when hourly air temperature (T, °C) is in the range between the minimum (TminCI, °C) and the maximum (TmaxCI, °C) for infection. Once CumLW and temperature are conducive, a moisture function [*f(M)*, 0-1, Eq. 5] triggers the infection events (*Infections*, 0-1, Eq. 7). A dry period, i.e., no leaf wetness, terminates the infection when its duration exceeds a threshold (LW50CI):


(5) f(M)={LWminCIf(T)     if     CumLW≥LWminCITminCI≤T≥TmaxCI0 otherwise

(6) $$f\left(T\right)=\left(\frac{TmaxCI-T}{TmaxCI-ToptCI}\right){\left(\frac{T-TminCI}{ToptCI-TminCI}\right)}^{\left(\frac{ToptCI-TminCI}{TmaxCI-ToptCI}\right)}$$

(7) Infections={1    if CumLW≥f(M)CumLW≥LWoptCI0otherwise

Where ToptCI is the optimum temperature for infection. Each infection event contributes increasing rotten incidence (Eq. 8). The parameter RottenCoefficient (Eq. 9) is modulated by the inoculum load from the previous year (*IncreaseR*, Eq. 3) and by the cumulated rainfall in January (CumR), normalized considering the median of the period 1984–2018 ($\bar{C\text{umR}}$):


(8) Rottenincidence=Infections*RottenCoefficient

(9) $$RottenCoefficient=IncreaseR+IncreaseR*\left(\frac{CumR-\bar{CumR}}{\bar{CumR}}\right)$$

The rotten model was developed as a BioMA component in Microsoft C#, following the guidelines of the Diseases components (Bregaglio and Donatelli, 2015; Valeriano et al., 2021; Wang et al., 2021).

### Sensitivity and Uncertainty Analysis

#### Analyzing the Weather Variability in the Study Area

The clusters of similarity in climatic conditions were identified computing agrometeorological indices (Table 1) in the period 1984–2018, centered in the hazelnut growing season (from October to October). Input data referred to each NASA-POWER grid cell in the Turkish hazelnut growing area.

**TABLE 1:** Agrometeorological indices computed on historical weather time series (1984–2018) in the Turkish hazelnut growing area.

Agrometeorological indices	Acronym	Unit of measure	Description	Ref.
Air frost yearly	AirFY	Days year^–1^	Number of days with *Tmin* <0°C	a
Rainfall yearly	RainY	mm year^–1^	Annual precipitation	b
Air temperature mean yearly	AirTMY	°C	Mean annual air temperature	b
Dryness	Dry	Days year^–1^	Number of days with precipitation <0.2 mm	a
Dry spell	DryS	Days year^–1^	Length of the longest dry period	b
Emberger continentality	EmbergerC	°C	Thermal excursion between the warmest and coldest month	c
Heat wave	HW	Days	Maximum number of consecutive days when *Tmax* >mean yearly *Tmax* + 3.0°C	a
Hot days number	HotDaysN	Days year^–1^	Number of days with *Tmax* >31°C	a
Mediterraneity	Med	Unitless	Ratio between the total precipitation in the warm and cold season. The higher the value, the more similar the climate to Mediterranean climate	d
Modified fournier	ModFournier	mm	Modified Fournier index calculated as the ratio between squared monthly precipitation and annual precipitation. The lower the value, the higher the dryness	e
Five days maximum rain amount	5DMaxRain	mm	Maximum total precipitation in 5 days	a
Wet spell	WS	Days year^–1^	Length of the longest rainy period	a
Desertification	Deser	Unitless	Desertification index calculated as the ratio between total annual precipitation and evapotranspiration. The lower the value, the higher the dryness	f

*Tmax: daily maximum air temperature (°C); Tmin: daily minimum air temperature (°C). References (Ref.): (a) Barnett et al., 2006; (b) basic statistics; (c) Emberger, 1930; (d) Le Houérou, 2004; (e) UNEP, 1992; (f) FAO/UNEP, 1977.*

A principal component analysis (PCA) was performed using these agrometeorological indices as active variables. Principal components (PCs) were obtained on centered and scaled variables, through the diagonalization of the correlation matrix and extraction of the associated eigenvectors and eigenvalues. A hierarchical clustering on principal components (HCPC) was then applied to identify the groups of years × NASA grid cells with similar climatic characteristics, using the Euclidean distance and Ward’s clustering algorithm (Giuliani et al., 2019).

For each cluster, ten NASA grid cells × year combinations were selected, i.e., the five most representative and the five most extreme considering the distance from their corresponding cluster centroid, to maximize the heterogeneity of the explored climatic variability. Simulations were performed on these situations, and results were aggregated at cluster level using mean and standard deviation (SD). A *v*-test (Lebart et al., 1995) was calculated on quantitative variables, under the null hypothesis (H0) that the cluster average did not differ from the overall average, with the sign of the test statistic indicating a lower (−) or greater (+) cluster mean than the overall mean. PCA and cluster analyses were performed using the FactoMineR R package (Husson et al., 2011).

#### Sensitivity Analysis Method

The sensitivity of the rotten simulation model to parameters variability was tested using the global sensitivity method by Sobol (1993) as improved by Saltelli (2002). This method is widely used in agroecological modeling studies, thanks to its robustness in identifying the parameters’ ranking and its capability of exploring the entire parameter space (DeJonge et al., 2012). This method decomposes the output variance into the terms of increasing dimension (i.e., partial variances), which represent the contribution of single parameters and of their combinations to the overall model outputs uncertainty (Eq. 10):


(10) $$D=\sum_{i=1}^{k}D_{i}+\sum_{1\leq i<j\leq k}D_{ij}+\ldots +D_{1,2,\ldots ,k}$$

Where *D* is the total output variance, *D*_*i*_ is the partial variance associated with the main effect of input factor (i), *D*_*ij*_ is the partial variance associated with the interaction between *i* and *j*, and *D*_1,2,..,_*_*k*_* is the interaction among *k* factors.

We used here the Sobol total sensitivity index (STI) to quantify the contribution of each parameter to output variability including all its interactions with other parameters (Homma and Saltelli, 1996). This index is computed as the sum of all sensitivity indices of different order (Eq. 11), which are calculated by dividing the partial variance of each parameter by the total variance of model outputs (*D*) (Eq. 12):


(11) $$ST_{i}=S_{i}\sum_{j\neq i}S_{i,j}+\ldots +S_{1,2,\ldots k}$$

(12) $$S_{i,j}=\frac{D_{i,j}}{D}$$

Where *S*_*i*_ provides the first-order contribution from the *i*-th input parameter to the output variance, *S*_*i,j*_ is the second-order contribution from the interaction between the *i*-th and the *j*-th parameters, and *S*_1,2,…_*_*k*_* is the contribution from the interaction among all *k* parameters.

The target outputs of the sensitivity analysis were the number of suitable hours for (a) pycnidia formation, (b) conidia spread, (c) infection, and (d) the rotten incidence (%). The latter was then directly compared with field samples in calibration and evaluation. All 16 parameters of the process-based models were included in the sensitivity analysis (Table 2). Given that no information is available on the thermal and moisture requirements of the *Diaporthe* spp. strains associated with hazelnut rotten defects, the default values of model parameters were set according to the biological requirements of *Phomopsis viticola*, i.e., anamorph of *Diaporthe viticola* and a causal agent of grapevine cane and leaf spot (Erincik et al., 2003; Anco et al., 2013). The random samples of model parameters were generated and 18,000 simulations were performed for each sensitivity analysis assessment. Simulation results were stored at daily temporal resolution and analyzed *via* boxplots to explore the uncertainty of the outputs. The sensitivity analysis was performed using the SALib package in Python (Herman and Usher, 2017).

**TABLE 2:** Acronym, units, and description of the parameters of the model of rotten incidence.

Parameter	Description	Units	Under calibration	Optimized/default value
HoursStartCS	Minimum number of consecutive hours with rainfall to trigger conidia spread	h	No	7
LW50CI	Dry hours to stop an infection event	h	No	5
LWminCI	Minimum leaf wetness duration to cause an infection	h	No	5
LWoptCI	Maximum leaf wetness duration to cause an infection	h	No	14
RainStartCS	Minimum rainfall amount to trigger conidia spread	mm	Yes	10.4
RHthresholdPF	Minimum relative humidity to trigger pycnidia formation	%	Yes	70
ThresholdPF	Sum of hourly temperature to trigger pycnidia formation	°C	No	152.3
TmaxCI	Maximum temperature to cause an infection	°C	No	32.4
TminCI	Minimum temperature to cause an infection	°C	No	6.4
ToptCI	Optimum temperature to cause an infection	°C	No	14.4
TminPF	Minimum temperature to trigger pycnidia formation	°C	Yes	9.6
TmaxPF	Maximum temperature to trigger pycnidia formation	°C	No	25.2
DVSstart	Phenological code to start the plant susceptibility period	Unitless	Yes	7.4
DVSend	Phenological code to end the plant susceptibility period	Unitless	Yes	10.9
DVSopt	Phenological code where plant susceptibility is maximum	Unitless	Yes	9.7
IP	Modulation of the increase of rotten due to the inoculum load from previous year	%	Yes	0.03

*Information on the inclusion in model calibration and calibrated value are also reported.*

### Model Calibration and Evaluation

The seven most relevant parameters explaining the variability of the outputs from the sensitivity analysis assessment were adjusted *via* automatic calibration, moving their values within their biological ranges (Table 2). The remaining 11 parameters were set as their default values (Table 2). Ground-truth data from the 22 orchards where weekly phenological observations in 2018–2019 and rotten incidence in 2016–2019 were available and were used for model calibration. The rotten incidence model was coupled with a multi-start downhill simplex algorithm (Nelder and Mead, 1965) to perform automatic calibration, setting a weighted root mean square error (RMSE, 0.5) and Pearson’s correlation coefficient (*r*, 0.5) as objective function to maximize both accuracy and correlations with reference data. We used 10 simplexes and 1,000 iterations, setting 0.001 as the tolerance value (Giuliani et al., 2019). After calibration, the model was applied on an independent dataset of 321 orchards, whose data were aggregated according to the respective NASA-POWER grid cell used as the source of weather data. Model performances were then assessed at municipality level, by taking the median of model results from all the NASA-POWER grid cells of interest. Model accuracy was assessed *via* mean absolute error (MAE, %), RMSE (%), Pearson’s *r*, and the coefficient of determination (*R*^2^).

## Results

### Sensitivity and Uncertainty Analysis

#### Assessing the Climatic Variability in the Study Area

**FIGURE 4:**
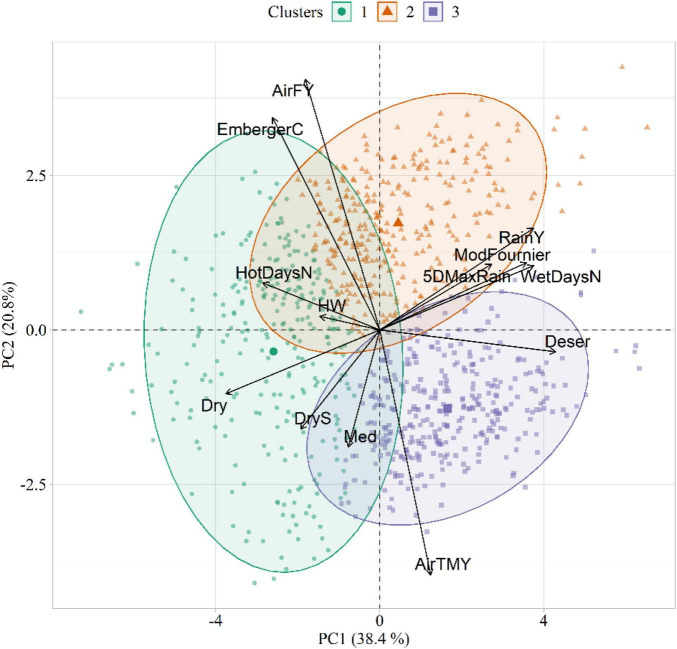
Biplot showing the agrometeorological indices in the principal component (PC) space, as well as the three clusters extracted in the first two PCs. The acronyms of the agrometeorological indices are reported in Table 1.

The results of the PCA and HCPC conducted on the agrometeorological indices are shown in Figure 4, whereas PCA loadings are reported in Table 3. The first two components, explaining 59% of the total variance, were selected for data interpretation. The first component (PC1) was mostly related to moisture conditions, as proved by its strong correlation with annual precipitation (RainY, *r* = 0.80), the number of wet days (WetDaysN, *r* = 0.80), the ratio between total annual precipitation and evapotranspiration (Deser, *r* = 0.92) and the Modified Fournier index (ModFournier, *r* = 0.77). The second component (PC2) depicted a thermal gradient, as it was positively correlated with mean yearly air temperature (AirTMY, *r* = −0.86), and negatively with the number of frost days (AirFY, *r* = 0.87).

**TABLE 3:** Principal component analysis (PCA) correlation and loadings of the first two principal components (PCs).

Index	Correlation		Loadings	
	PC1	PC2	PC1	PC2
5DMaxRain	0.59	0.23	0.26	0.14
AirFY	–0.39	0.88	–0.18	0.53
AirTMY	0.27	–0.86	0.12	–0.52
Deser	0.93	–0.08	0.41	–0.05
Dry	–0.81	–0.22	–0.36	–0.14
DryS	–0.42	–0.35	–0.19	–0.21
EmbergerC	–0.56	0.74	–0.25	0.45
HotDaysN	–0.62	0.16	–0.28	0.10
HW	–0.32	0.05	–0.14	0.03
Med	–0.17	–0.41	–0.07	–0.25
ModFournier	0.77	0.24	0.35	0.14
RainY	0.81	0.36	0.36	0.22
WetDaysN	0.81	0.22	0.36	0.14

*The acronyms of the agrometeorological indices are explained in Table 1.*

The HCPC identified three clusters of similarities in climatic conditions (Figure 4). Cluster 1 (C1) was characterized by dry weather conditions and high daily thermal excursion (average maximum and minimum temperature equal to 16.5 and 5.3°C, respectively). The main representative agrometeorological indices in the characterization of this cluster were Dry (*v* = 18.89, *p* < 0.001), HotDaysN (*v* = 15.63, *p* < 0.001), EmbergerC (*v* = 11.59, *p* < 0.001), DryS (*v* = 11.29, *p* < 0.001), and HW (*v* = 7.25, *p* < 0.001). Cluster 2 (C2) identified years with the highest amount of average annual rainfall (823 mm). This cluster corresponded to cold temperatures with high daily excursion, with maximum and minimum temperature equal to 14.3 and 6.0°C, respectively. The main representative agrometeorological indices in C2 were AirFY (*v* = 11.59, *p* < 0.001), RainY (*v* = 12.42, *p* < 0.001), and EmbergerC (*v* = 12.33, *p* < 0.001). Cluster 3 (C3) emerged as a representative of a warm and wet environment (average annual rainfall = 792 mm). Differently from C1 and C2, this cluster was characterized by a narrow daily thermal excursion (16.17 and 11.85°C for maximum and minimum temperature, respectively). The main representative agrometeorological indices in C3 were AirTMY (*v* = 20.96, *p* < 0.001), Deser (*v* = 17.86, *p* < 0.001), and WetDaysN (*v* = 10.01, *p* < 0.001). The *v*-tests results for all active variables in the three clusters are listed in Supplementary Tables 3–5.

#### Exploring Model Plasticity in Different Climatic Conditions

**FIGURE 5:**
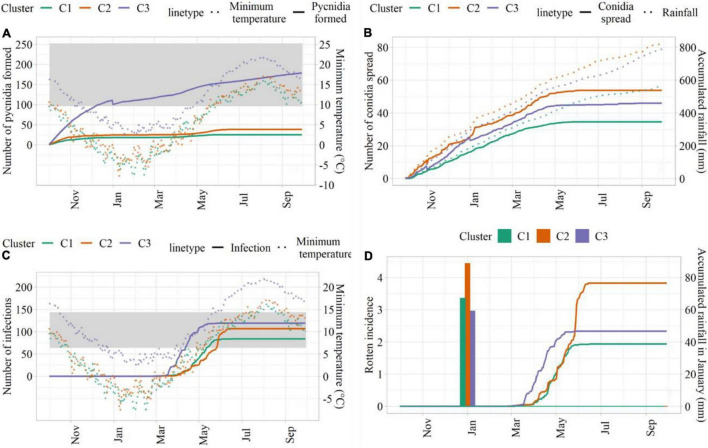
Model outputs from sensitivity analysis. **(A)** Hours for pycnidia formation (solid lines) and minimum temperature (°C; dotted lines); **(B)** hours of conidia spread (solid lines) and cumulated rainfall (dotted lines); **(C)** infection hours (solid lines) and minimum temperature (°C; dotted lines); **(D)** simulated rotten incidence (%, solid lines) and cumulated rainfall in January (bars). The ranges of suitable temperature are indicated as shades in panels **(A,C)**.

Simulations conducted with weather data from C3 led to the highest number of suitable hours for pycnidia formation, followed by C2 and C1 (Figure 5A). This was mainly due to the higher minimum temperature in C3, which led to the longer favorable period for pycnidia formation. The number of hours, when conidia spread was simulated, was higher in C2, followed by C3 and C1 (Figure 5B), in agreement with larger precipitation amounts. C3 led to the highest number of infection hours (Figure 5C), mainly due to more favorable thermal conditions. Simulated rotten incidence was higher in C2 (3.82%), followed by C3 (2.33%) and C1 (1.94%, Figure 5D). As described in section “Model Development,” the simulated rotten incidence is affected by the number of infection events during the growing season and modulated by cumulated rainfall in January, which was higher in C2 (88.6 mm). C3, on the other hand, was associated with the lowest cumulated rainfall in January, but led to higher rotten incidence than C1 due to the higher number of simulated infections.

**FIGURE 6:**
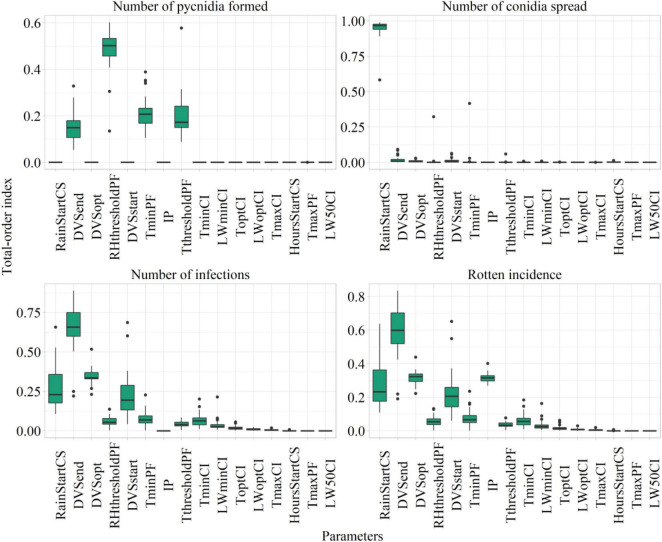
Sobol total sensitivity index (STI) from Sobol sensitivity analysis considering main model outputs. The description of the parameters is presented in Table 3.

The values of Sobol total-order index computed on model parameters after sensitivity analysis are presented as a boxplot in Figure 6, considering the number of suitable hours for pycnidia formation, conidia spread, infection, and the rotten incidence.

The most relevant parameters from the sensitivity analysis were subjected to automatic calibration. They were the phases of start, maximum, and end susceptibility (DVSstart, STI = 0.22 ± 0.13; DVSopt, STI = 0.31 ± 0.04; and DVSend, STI = 0.58 ± 0.14); the rainfall amount triggering conidia spread (RainStartCS, STI = 0.26 ± 0.13); the minimum temperature and threshold of air relative humidity for pycnidia formation (TminPF, STI = 0.07 ± 0.05; RHthresholdPF, STI = 0.05 ± 0.03); and the maximum increase of rotten incidence (IP, STI = 0.31 ± 0.03).

The RHthresholdPC and TminPF have mostly contributed to the variability in the number of suitable hours for pycnidia formation, given that this process is mainly driven by temperature and relative humidity. RainStartCS was the most relevant parameter influencing the number of suitable hours for conidia spread, which are entirely dependent on rainfall. The number of favorable hours for infections resulted mainly dependent on IP and plant susceptibility, as proved by the top-ranked parameters, which were the ones related to the period of plant susceptibility.

### Rotten Simulation Model Calibration and Evaluation

Model performances in calibration are reported in Table 4 as aggregated to the municipality level, considering the location of the 22 orchards where weekly phenological observations and rotten incidence data were available (Figure 2). In the Western Black Sea region, RMSE in predicting rotten incidence was always below 2.00%, and MAE ranged between 0.50% in Duzce and 1.40% in Zonguldak. Pearson’s correlation coefficient was higher than 0.50 in all municipalities, even if significant values were reached only in Sakarya, due to the low number of orchards in each municipality. In the Eastern Black Sea region, model accuracy was slightly lower, with RMSE in predicting rotten incidence between 1.26% in Trabzon and 2.55% in Samsun, the latter municipality leading to a MAE higher than 2.00%. Trabzon was the only municipality where Pearson’s correlation coefficient was significant. However, when considering all orchards in the same region, Pearson’s *r* was significant both in Western (*r* = 0.72) and Eastern (*r* = 0.45) region, as well as when evaluation metrics were computed on the whole calibration dataset. Model performances in calibration at the country level denoted a good model accuracy (RMSE = 1.26% and MAE = 0.99%) and a significant correlation (*r* = 0.58) with field observations.

**TABLE 4:** Model performances in reproducing rotten incidence in calibration.

Geographical area	RMSE	MAE	Pearson correlation
**West**			
Duzce	0.81	0.50	0.79^ns^
Sakarya	0.71	0.57	0.93*
Zonguldak	1.70	1.40	0.58^ns^
Mean	1.15	0.82	0.72*
**East**			
Giresun	2.21	1.77	0.69^ns^
Ordu	2.21	1.68	0.47^ns^
Samsun	2.55	2.24	0.19^ns^
Trabzon	1.26	0.99	0.89*
Mean	2.11	1.66	0.45*
Turkey	1.77	1.31	0.58*

The evaluation metrics of model accuracy are synthesized for the whole country and are reported at regional and municipality level. ^ns^p > 0.05; *p < 0.05.

**FIGURE 7:**
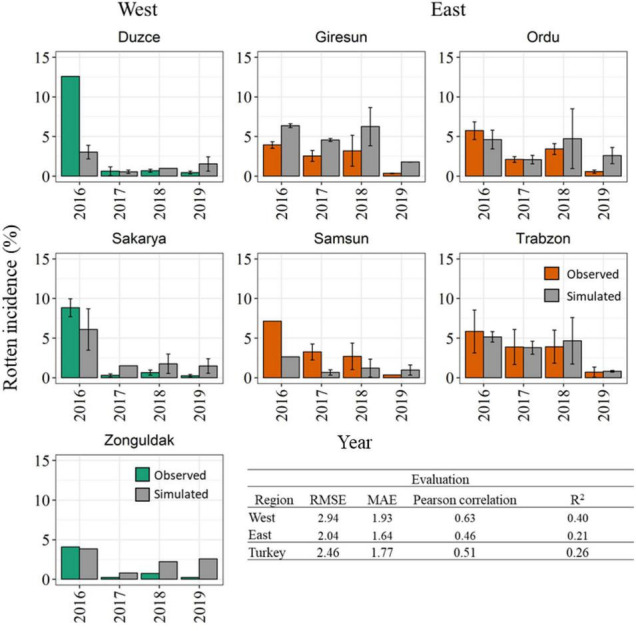
Comparison between simulated (gray bars) and observed rotten incidence (%) in 2016–2019 in the seven main hazelnut growing municipalities in the Western (green bars) and Eastern (orange bars) Black Sea region. The variability associated with field surveys are presented by the standard error (SE) (error bars). Statistical metrics quantifying the model accuracy in the evaluation dataset are reported at country and regional level.

The model accuracy in reproducing reference data coming from an independent dataset of 321 additional orchards are presented in Figure 7. The histogram analysis highlights that the model correctly reproduced a very high rotten incidence in 2016 in the Western Black Sea region, even if the exceptional rotten incidence in Duzce (>13.00%) was underestimated. Coherently with field observations, the model simulated lower rotten incidence in the period of 2017–2019 in all Western municipalities, with average model errors lower than 2.00% in all cases. In the Eastern Black Sea region, the model was able to reproduce an overall higher rotten incidence than in the Western region, as well as to match the higher observed rotten incidence in 2016–2018, especially in Giresun, Ordu, and Trabzon, whereas in Samsun the model underestimated the reference data. Simulations in 2019 highlighted low rotten incidence in all the municipalities of the Eastern region, in agreement with field data. The evaluation metrics computed on the validation dataset denoted slightly lower performances than in calibration, with RMSE comprised between 2.04% in the Eastern and 2.94% in the Western Black Sea region, and MAE lower than 2.00% in all cases. Pearson’s correlation coefficient computed at municipality level ranged between 0.46 in the Eastern and 0.63 in the Western Black Sea, with corresponding *R*^2^ of 0.26 at country level.

## Discussion

One of the main determinants of hazelnuts price is the incidence of rotten defect, a major concern for the confectionary industry. The identification of its causal agents has been debated in the last years. Recently, *Diaporthe* spp. were identified as candidate etiological pathogens in the Caucasian region by Battilani et al. (2018) and in Turkey by Arciuolo et al. (2020). The rationale behind the development of the process-based model presented here lays the pieces of evidence from these studies, which found a significant correlation between *Diaporthe* spp. and the incidence of rotten defect in similar hazelnut cultivation areas. However, the same authors found other fungal species associated with damaged kernels, and new field experiments and molecular analyses are ongoing to gain new insights on the actual etiology of this defect. On these premises, we decided to target the main components of the epidemiological cycle of *Diaporthe* spp. using a generic modeling approach, which is grounded on the *Diaporthe* lifecycle, but also customizable to other pathogens both *via* parameterization or the substitution of specific (sub-) models when new information is available.

The workflow for the model development comprised an uncertainty and sensitivity analysis to rank the most relevant parameters in modulating model outputs, whose results were used as input for an automatic model calibration, using in-field rotten incidence as reference data (Bregaglio et al., 2020). The cardinal optimum, maximum, and minimum temperature for infection and latency were set to 14, 32, and 6°C, respectively. This agrees with Erincik et al. (2003) and Anco et al. (2013), who analyzed temperature and wetness duration requirements for grape leaf and cane infection by *P. viticola* (same genus as *Diaporthe*). The positive correlation between the incidence/severity of plant fungal diseases and warm and humid weather conditions is well known (Rotem and Palti, 1969; Colhoun, 1973; Café-Filho et al., 2019). The uncertainty analysis highlighted that a wet and warm environment with a small temperature variability (Cluster 3) is more suitable for the infection process. This result can be mostly attributed to more humid conditions in C3, which was also associated with small limitations in thermal requirements for fungal infections. Anco et al. (2013) stated that once sporulation occurs, infections would also follow shortly thereafter, provided that the leaf wetness lasts and temperature does not drastically change. Therefore, the other two clusters from the HCPC analysis, where temperature variability was larger, were associated with higher limitations to the fulfillment of plant susceptibility, and in turn, the number of suitable hours for infection.

The main purpose of the sensitivity analysis was the identification of parameters with high influence on the variability of the outputs (Specka et al., 2019), to select a subset of parameters for the subsequent calibration (Makowski et al., 2011). The rotten simulation model showed to be sensitive to parameters related to plant susceptibility, with DVSend accounting for the highest variability in simulated rotten incidence. This underlines the importance of the accurate simulation of hazelnut reproductive phenology, given that the plant is mostly susceptible to fungal infections during female flowering. The simulation model was able to reproduce the observed trends of rotten incidence in the main hazelnut areas in Turkey, and to simulate the highest rotten incidence in 2016. These results agree with Battilani et al. (2018), who noticed severe symptoms of black, completely decayed rotten nuts in 2016 in the Caucasus region.

Overall, inter-annual variability of the rotten incidence was adequately reproduced by the model, which responded to environmental conditions, such as rainfall and temperature (Erincik et al., 2003; Huber et al., 2006; Nita et al., 2008). In particular, rainfall is crucial for fungal dissemination (Erincik et al., 2003). In addition, Battilani et al. (2018) reported large precipitation amount in 2016 in the Caucasian region. However, the model validation revealed that, in its current form, the model partially failed in reproducing exceptionally high values of rotten incidence (e.g., in Duzce, 2016). The huge variability of rotten incidence within the same growing season in the validation dataset, constituted by 321 additional sampling locations, denote a major effect of agronomic and pedo-climatic conditions that are not currently take into account in the predictive workflow. This is the main reason contributing to explain the low correlation of reference and simulated rotten incidence in the Eastern Black Sea region, where hazelnut orchards management techniques are generally less advanced and soil conditions are much more variable than in the Western region (Erdogan, 2018). Other reasons which could contribute to explain this poor agreement with field observations lie in instrumental, procedural, and human errors in laboratory analyses. The most important limitation, however, is the spatial resolution of the input gridded weather data, which were available at a granularity of 0.5° × 0.5° resolution, corresponding to approximately 3,000 km^2^ on the ground. This study would have been benefited from the availability of either gridded weather datasets at a higher resolution (e.g., ERA5-Land from Copernicus), or from weather stations placed close to the fields, which would have allowed us to gain more insights into the meso- and microclimatic conditions especially of the Eastern Black Sea area. We highlight that the model presented here is in a prototypal form and would benefit from a set of detailed laboratory experiments to test the response of epidemiological processes to the varying moisture/thermal conditions, as well as from field trials where alternative management practices and weather conditions are tested *ad hoc* to extrapolate specific response functions. The availability of additional knowledge on the actual etiology of rotten hazelnuts coming from laboratory and field experiments will be fundamental to revise part of the modeling framework presented in this paper, by *ad hoc* functions targeting key components of the epidemiological cycle of specific fungal pathogens. Eventually, the model performances shall be evaluated in other hazelnut cultivation regions, to assess the model scalability in predicting rotten incidence in different environments.

## Conclusion

The reliable process-based models to predict quality defects caused by plant pathogens are requested to the modeling community. The model presented here is the first building block of a decision support system to support the tactical decisions of plant protection in hazelnut orchards, such as the application of agrochemicals, and to forecast the expected impact of rotten incidence at the end of the growing season. The operational execution of this model will require the use of seasonal weather forecasts as model input to anticipate expected trends in rotten hazelnuts some weeks before harvest. The spatially distributed application of the model in the main hazelnut producing municipalities of Turkey is a preliminary step toward the extension of the same procedure to other environments, provided that sufficient input data are available. Such possibility will open new perspectives for hazelnuts buyers, who need support to optimize their purchase strategies in the different regions of the globe.

## Data Availability Statement

The raw data supporting the conclusions of this article will be made available by the authors upon reasonable request.

## Author Contributions

SB and TV conceived and wrote the rotten hazelnuts prediction model, performed the principal components and sensitivity analysis, analyzed model results, and wrote the draft version of the manuscript. KF and LG supervised the project and guide the development of the rotten hazelnut prediction model. FG supported the climatic analysis and the automatic model calibration. GC shared the ground truth data on rotten incidence and provided domain expert knowledge to develop the simulation activities. All authors contributed to the article writing and approved the submitted version.

## Conflict of Interest

TV and KF were employed by the company Ferrero Hazelnut Company, Ferrero Trading Lux S.A. LG was employed by the company RSS-Hydro SARLS. GC was employed by the company SOREMARTEC ITALIA S.r.l. The remaining authors declare that the research was conducted in the absence of any commercial or financial relationships that could be construed as a potential conflict of interest.

## Publisher’s Note

All claims expressed in this article are solely those of the authors and do not necessarily represent those of their affiliated organizations, or those of the publisher, the editors and the reviewers. Any product that may be evaluated in this article, or claim that may be made by its manufacturer, is not guaranteed or endorsed by the publisher.
