# Supplementary material for: Rotten Hazelnuts Prediction via Simulation Modeling—A Case Study on the Turkish Hazelnut Sector
Source: Front Plant Sci. 2022 Apr 4;13:766493. doi: 10.3389/fpls.2022.766493 (PMC9014268; doi:10.3389/fpls.2022.766493)
Supplement: Supplementary file 1 [file Table_1.DOCX]

**Supplementary materials**

WKDGp7co52Y1M3qbbNf+BpPxvx7loGoFs9qRIk9MYHVmZ3I4et9LYGnOYKr7T7y7rVQP4kD5rptzEJb7ujIjEAVjVEtw4qKNo5w8vXL0ZHhcYIWsnDi+UHMma+38tGtbASVgLa76kELoYwbzSaqEXASIJKhY0FpHsXp/olsPmTw=

Table S1. Sampled orchards and the main characteristics.

| Orchard | Region | Municipality | Latitude | Longitude | Altitude | Main variety |
| --- | --- | --- | --- | --- | --- | --- |
| Orchard01 | West | Sakarya | 40.76 | 30.72 | 197.00 | Çakıldak |
| Orchard02 | West | Duzce | 41.06 | 31.03 | 112.00 | Mincane |
| Orchard03 | West | Duzce | 40.89 | 30.85 | 600.10 | Çakıldak |
| Orchard04 | West | Duzce | 40.77 | 30.96 | 139.80 | Mincane |
| Orchard05 | East | Giresun | 41.04 | 39.09 | 60.80 | Tombul |
| Orchard06 | East | Ordu | 40.84 | 37.78 | 413.60 | Çakıldak |
| Orchard07 | East | Giresun | 40.88 | 38.18 | 343.70 | Tombul |
| Orchard08 | West | Sakarya | 40.99 | 30.75 | 243.00 | Çakıldak |
| Orchard09 | East | Trabzon | 40.95 | 39.99 | 106.90 | Foşa |
| Orchard10 | East | Ordu | 40.97 | 37.86 | 156.40 | Palaz |
| Orchard11 | West | Zonguldak | 41.10 | 31.41 | 322.40 | Mincane |
| Orchard12 | West | Sakarya | 40.96 | 30.87 | 489.40 | Çakıldak |
| Orchard13 | West | Duzce | 40.95 | 31.24 | 684.40 | Çakıldak |
| Orchard14 | East | Trabzon | 40.95 | 39.67 | 617.70 | Sivri |
| Orchard15 | East | Trabzon | 40.83 | 39.63 | 369.60 | Mincane |
| Orchard16 | East | Ordu | 40.88 | 37.88 | 346.00 | Kara Fındık |
| Orchard17 | East | Ordu | 41.09 | 37.74 | 397.40 | Palaz |
| Orchard18 | East | Samsun | 40.98 | 36.61 | 454.70 | Çakıldak |
| Orchard19 | East | Ordu | 40.82 | 37.62 | 1010.30 | Çakıldak |
| Orchard20 | East | Giresun | 40.84 | 38.64 | 424.40 | Tombul |
| Orchard21 | East | Samsun | 41.29 | 36.77 | 10.00 | Çakıldak |
| Orchard22 | East | Samsun | 41.12 | 36.88 | 95.80 | Palaz |

Table S2. Codes and description of the vegetative and reproductive phenological phases simulated by the hazelnut modelling solutions according to Bregaglio et al. (2016, 2020, 2021).

| Type | DVS code | Description |
| --- | --- | --- |
| Reproductive  (male flowers) | R1 | Catkins emerge |
|  | R2 | Catkins full developed but dormant |
|  | R3 | Catkins begin to elongate |
|  | R4 | Catkins in full bloom |
|  | R5 | Catkins withering |
| Reproductive  (female flowers) | R7 | Beginning of female flowering |
|  | R8 | Inflorescence in full bloom |
|  | R9 | End of female flowering |
|  | R10 | Ovaries develop |
|  | R11 | Cluster visible |
|  | R12 | Immature fruits |
|  | R13 | Fruits maturation |
|  | R14 | Fruits dropping |
|  | R15 | All nuts dropped |
| Vegetative | V1 | Dormant bud |
|  | V2 | Swollen bud |
|  | V3 | Budbreak |
|  | V4 | Leaf emergence |
|  | V5 | 3^rd^ leaf unfolded |
|  | V6 | Mature leaves are spotted |
|  | V7 | Mature leaves |
|  | V8 | Leaf senescence |
|  | V9 | Leaf shedding |
|  | V10 | Leaves dropping |

Table S3. Description of Cluster 1 by quantitative variables.

| Agrometeorological indices | v.test | Mean in category | Overall mean | Standard deviation in category | Overall standard deviation | p.value |
| --- | --- | --- | --- | --- | --- | --- |
| Dry | 18.89 | 197.02 | 180.81 | 15.57 | 19.17 | 0.00 |
| HotDaysN | 15.63 | 8.87 | 4.13 | 8.83 | 6.77 | 0.00 |
| EmbergerC | 11.59 | 29.63 | 27.39 | 3.63 | 4.32 | 0.00 |
| DryS | 11.29 | 20.53 | 16.82 | 6.46 | 7.33 | 0.00 |
| HW | 7.26 | 1.10 | 0.41 | 3.44 | 2.13 | 0.00 |
| AirFY | 6.87 | 63.55 | 50.96 | 33.98 | 40.95 | 0.00 |
| Med | 4.86 | 2.26 | 2.03 | 1.35 | 1.08 | 0.00 |
| AirTMY | -2.95 | 11.92 | 12.19 | 1.54 | 2.01 | 0.00 |
| 5DMaxRain | -15.47 | 46.95 | 59.14 | 11.74 | 17.61 | 0.00 |
| ModFournier | -18.70 | 732.51 | 894.72 | 130.83 | 193.85 | 0.00 |
| WetDaysN | -18.83 | 166.46 | 182.56 | 15.60 | 19.10 | 0.00 |
| RainY | -19.09 | 556.06 | 684.45 | 95.15 | 150.34 | 0.00 |
| Deser | -21.40 | 4.99 | 7.10 | 0.94 | 2.20 | 0.00 |

Table S4. Description of Cluster 2 by quantitative variables.

| Agrometeorological indices | v.test | Mean in category | Overall mean | Standard deviation in category | Overall standard deviation | p.value |
| --- | --- | --- | --- | --- | --- | --- |
| AirFY | 18.00 | 89.48 | 50.96 | 22.19 | 40.95 | 0.00 |
| RainY | 12.43 | 782.13 | 684.45 | 142.13 | 150.34 | 0.00 |
| EmbergerC | 12.33 | 30.18 | 27.39 | 2.41 | 4.32 | 0.00 |
| ModFournier | 9.70 | 993.05 | 894.72 | 176.87 | 193.85 | 0.00 |
| WetDaysN | 9.17 | 191.71 | 182.56 | 14.47 | 19.10 | 0.00 |
| 5DMaxRain | 8.64 | 67.09 | 59.14 | 18.06 | 17.61 | 0.00 |
| Deser | 3.42 | 7.49 | 7.10 | 1.61 | 2.20 | 0.00 |
| HW | -3.40 | 0.03 | 0.41 | 0.43 | 2.13 | 0.00 |
| HotDaysN | -3.87 | 2.76 | 4.13 | 4.20 | 6.77 | 0.00 |
| Med | -7.27 | 1.62 | 2.03 | 0.65 | 1.08 | 0.00 |
| Dry | -9.22 | 171.58 | 180.81 | 14.53 | 19.17 | 0.00 |
| DryS | -10.11 | 12.95 | 16.82 | 5.92 | 7.33 | 0.00 |
| AirTMY | -19.49 | 10.15 | 12.19 | 1.28 | 2.01 | 0.00 |
|  |  |  |  |  |  |  |

Table S5. Description of Cluster 3 by quantitative variables.

| Agrometeorological indices | v.test | Mean in category | Overall mean | Standard deviation in category | Overall standard deviation | p.value |
| --- | --- | --- | --- | --- | --- | --- |
| AirTMY | 20.96 | 13.96 | 12.19 | 1.00 | 2.01 | 0.00 |
| Deser | 17.86 | 8.76 | 7.10 | 1.80 | 2.20 | 0.00 |
| WetDaysN | 10.01 | 190.59 | 182.56 | 15.05 | 19.10 | 0.00 |
| ModFournier | 9.38 | 971.22 | 894.72 | 154.29 | 193.85 | 0.00 |
| RainY | 7.24 | 730.20 | 684.45 | 111.69 | 150.34 | 0.00 |
| 5DMaxRain | 7.19 | 64.47 | 59.14 | 15.65 | 17.61 | 0.00 |
| Med | 1.97 | 2.12 | 2.03 | 0.95 | 1.08 | 0.05 |
| DryS | -1.72 | 16.29 | 16.82 | 7.38 | 7.33 | 0.09 |
| HW | -3.98 | 0.05 | 0.41 | 0.64 | 2.13 | 0.00 |
| Dry | -10.02 | 172.73 | 180.81 | 15.10 | 19.17 | 0.00 |
| HotDaysN | -11.77 | 0.78 | 4.13 | 2.00 | 6.77 | 0.00 |
| EmbergerC | -22.82 | 23.25 | 27.39 | 2.39 | 4.32 | 0.00 |
| AirFY | -23.44 | 10.60 | 50.96 | 12.63 | 40.95 | 0.00 |
|  |  |  |  |  |  |  |
